# Neostigmine for non-mild acute pancreatitis: A systematic review and meta-analysis of randomized controlled trials

**DOI:** 10.3389/fphar.2023.1131974

**Published:** 2023-02-28

**Authors:** Kun He, Yabing Wang, Jianing Li, Xiaoyin Bai, Zihan Yang, Xianlin Han, Dong Wu

**Affiliations:** ^1^ Department of Gastroenterology, State Key Laboratory of Complex Severe and Rare Diseases, Peking Union Medical College Hospital, Chinese Academy of Medical Sciences and Peking Union Medical College, Beijing, China; ^2^ Department of Endocrinology, Beijing Friendship Hospital, Capital Medical College, Beijing, China; ^3^ Department of General Surgery, Peking Union Medical College Hospital, Peking Union Medical College, Chinese Academy of Medical Sciences, Beijing, China; ^4^ Clinical Epidemiology Unit, Peking Union Medical College Hospital, Peking Union Medical College, Chinese Academy of Medical Sciences, Beijing, China

**Keywords:** acute pancreatitis, neostigmine, prokinetic drug, gastrointestinal motility, prognosis

## Abstract

**Background:** The therapeutic value of neostigmine as a prokinetic drug in acute pancreatitis (AP), especially in non-mild AP, including moderately severe and severe AP remains controversial. This meta-analysis aimed to investigate the efficacy of neostigmine treatment in patients with non-mild AP.

**Methods:** We searched the Cochrane Central Register of Controlled Trials (CENTRAL), MEDLINE, Embase, China National Knowledge Infrastructure (CNKI), and Wanfang databases up to 24 December 2022 for RCTs comparing neostigmine plus conventional treatment versus the conventional treatment alone in patients with non-mild AP. Trial sequential analyses (TSA) were used to assess the risk of random errors and the results.

**Results:** Six RCTs with 318 participants were included. Compared with conventional treatment, patients who received neostigmine plus conventional treatment had a shorter time duration for their first defecation (MD: −1.74; 95% CI: −2.10 to −1.38; *p* < 0.00001; *n* = 205; RCTs = 4; low quality of evidence) and better relief time of abdominal symptoms (MD: −1.59, 95% CI: −2.07 to −1.11; *p* < 0.00001; *n* = 155; RCTs = 3; low quality of evidence) as primary outcomes, and a faster percentage decrease of IAP at 24 h (*p* = 0.0005; moderate quality of evidence) and a shorter length of ICU stay (*p* < 0.00001; moderate quality of evidence) as partial secondary outcomes. TSA suggested the sample size was limited, but the cumulative Z curves of the primary outcomes crossed the conventional boundary and the trial sequential monitoring boundary.

**Conclusion:** For patients with non-mild AP, neostigmine promotes the recovery of gastrointestinal motility and may have positive effects on the improvement of a clinical prognosis. Further large-sample studies are needed for a definite conclusion.

**Systematic Review Registration:** https://www.crd.york.ac.uk/prospero/; Identifier: CRD 42022381417.

## Introduction

Acute pancreatitis (AP) is acute inflammation of the pancreas with a variable involvement of the nearby tissues or other organs, causing substantial mortality and morbidity (Bradley, 1993; Boxhoorn et al., 2020). It is one of the most common causes for gastroenterology-related hospitalization, with an increase in the annual incidence of AP in the past decades (Peery et al., 2012; Roberts et al., 2013). Based on the presence of organ failure and local and systemic complications, AP was classified into mild AP (MAP) and non-mild AP, including moderately severe AP (MSAP) and severe AP (SAP), according to the revised Atlanta Classification. MAP presents with a self-limitation process, but non-mild AP accounts for substantial morbidity and mortality and needs aggressive treatment (Banks et al., 2013).

Gut dysfunction, with ileus as the most frequently encountered complication, is common in AP, especially SAP (Uhl et al., 1996; Wang et al., 2003). It often aggravates intra-abdominal hypertension (IAH) and may even give rise to abdominal compartment syndrome (ACS) (Kirkpatrick et al., 2013; van Brunschot et al., 2014). In addition, gut dysfunction also limits the starting and delivery of enteral nutrition, which has been confirmed to reduce infections, surgical interventions, organ failures, and mortality (Cao et al., 2008; Al-Omran et al., 2010). Therefore, the concept of ‘gut rousing’ was proposed to maintain the gut function, while there was no effective evidence-based drugs except for supportive treatment in clinical practice until now (Petrov and Windsor, 2013; Moggia et al., 2017).

Neostigmine is an anti-cholinesterase drug that enhances intestinal peristalsis, promoting the passage of flatus and defecation. It is widely used as a prokinetic drug in the treatment of AP, especially with gut dysfunction and IAH. Several previous studies showed that it had positive effects on AP with the latest randomized controlled trials (RCTs) published in March 2022, suggesting that neostigmine could reduce IAH and promote defecation (Schneider et al., 2014; He et al., 2022; Mancilla Asencio and Berger Fleiszig, 2022). However, its clinical value for AP has remained debatable so far, and the current international clinical guidelines did not attribute enough importance to this drug. To date, no systemic review has addressed this topic either (Tenner et al., 2013; Greenberg et al., 2016; Crockett et al., 2018a; Leppaniemi et al., 2019). Therefore, we planned to conduct a systematic review and meta-analysis to explore the efficacy of neostigmine for the treatment of non-mild AP, aiming to provide current evidence for clinical practice.

## Methods

This study was conducted based on the Preferred Reporting Items for Systematic Reviews and Meta-Analyses (PRISMA) protocol (Supplementary Table S1, PRISMA checklist) and the recommendations of the Cochrane Handbook for Systematic Reviews of Interventions (The Cochrane Collaboration, 2011; Page et al., 2021). The study protocol was prospectively registered on PROSPERO (CRD 42022381417).

## Eligibility criteria

### Study types

Our study only included RCTs, while other types of studies such as case series, case reports, and observational cohort studies were excluded. Studies without sufficient data, primary data, or full text were also excluded, and neither were duplicate publications. Languages were not limited.

### Participants

Our study included adults (aged over 18) with non-mild AP including MSAP and SAP, and participants with contraindications to neostigmine, such as an allergy to neostigmine, comorbidity with epilepsy, angina pectoris or asthma, and pregnancy or lactation, were excluded (Neely et al., 2022). MSAP was defined as AP that had local complications with or without transient organ failures (<48 h). SAP was characterized by persistent organ failure (>48 h) with or without local complications. The diagnosis of AP and definitions of MSAP and SAP were made according to the established guidelines (Tenner et al., 2013; Greenberg et al., 2016; Crockett et al., 2018a; Leppaniemi et al., 2019; Chinese Pancreatic Surgery Association CSoSCMA, 2021).

### Interventions and comparisons

We planned to include studies comparing neostigmine plus conventional therapy as the neostigmine treatment (NT) group and conventional therapy alone as the conventional treatment (CT) group. Conventional therapy included fluid management with early fluid resuscitation and volume control for IAH, analgesics, nutrition support, symptomatic treatment, gastrointestinal decompression, and traditional Chinese medicine (TCM) components such as rhubarb, Glauber’s salt, and Da-Cheng-Qi decoction (Qiong et al., 2005; Boxhoorn et al., 2020). Studies with different kinds of conventional treatment between NT and CT were excluded. The route of neostigmine included intramuscular injections and Zusanli (stomach meridian, ST36), which is an acupoint 2 cm below the knee joint on the anterior aspect of the lower limb based on the TCM theory of acupuncture and had potential effects on the recovery of gastrointestinal disorders (Ng et al., 2013).

## Outcomes

Primary outcomes: a. Time to the first defecation; b. time to the relief of abdominal symptoms.

Secondary outcomes: a. Percentage decrease of intra-abdominal pressure (IAP) at 24 h; b. new-onset ACS that is defined as a sustained IAP>20 mmHg with organ failure after treatment (Kirkpatrick et al., 2013); c. in-hospital mortality; e. multiple organ failure; d. interventional drainage and operation events; e. length of ICU stay; f. length of hospital stay; and g. serious adverse events caused by neostigmine, which include nervous system dysfunctions such as ataxia, convulsions, coma, slurred speech, anxiety or fear, malignant arrhythmia, or bronchospasm (Smedley et al., 2020).

## Search methods

We conducted a literature search up to 24 December 2022 in the Cochrane Central Register of Controlled Trials (CENTRAL), MEDLINE, Embase, China National Knowledge Infrastructure (CNKI), and Wanfang databases with the designed search strategies (Supplementary Table S2). The reference lists of relevant articles were also checked for additional references.

## Data collection and analyses

### Study selection and data extraction

Two reviewers (JL and XB) independently completed study screening and selection. First, the reviewers screened the titles and abstracts of potential studies from the literature search and retrieved the qualified articles. Afterward, the reviewers screened the full text and identified eligible studies for the inclusion based on inclusion and exclusion criteria. Disagreements were resolved through discussions. In addition, we identified and excluded duplicates and multiple reports of the same study. Another set of reviewers (YW and ZY) independently conducted data extraction and recorded all relevant details from the included studies using a standardized data extraction form including methods, participants, interventions, outcomes, and additional related information. Similarly, disagreements were resolved through discussions.

### Assessment of the risk of bias in the included studies and certainty of evidence

Two reviewers (XB and ZY) independently assessed the risk of bias for each study and recorded it in ‘Risk of bias’ tables with conflicts resolved through discussions. The risk of bias of RCTs was assessed with items in the Cochrane Collaboration’s tool (The Cochrane Collaboration, 2011).

We applied the Grading of Recommendations Assessment, Development, and Evaluations (GRADE) framework to assess the certainty of evidence on the main outcomes, including primary outcomes and several important secondary outcomes, such as the time to the first defecation, time to the relief of abdominal symptoms, percentage decrease of IAP at 24 h, and the length of ICU stay (Atkins et al., 2004).

### Trial sequential analyses

Trial sequential analyses (TSA) were used to control the risk of random errors and assess the conclusions. Based on previous clinical experiences and RCTs in this field, we used the mean difference (MD) and a variance of power of 80% to calculate the required sample size and assess the clinical significance of the primary outcome in our review (He et al., 2022). Decisions were made based on the position of cumulative Z curves with the conventional boundary, trial sequential monitoring boundary, and futility boundary (Wetterslev et al., 2017).

### Assessment of heterogeneity

We utilized the I^2^ statistic to measure the heterogeneity among the RCTs. We considered an I^2^ value greater than or equal to 60% as the evidence of moderate to substantial levels of heterogeneity. In the event of I^2^ > 80% (substantial heterogeneity), we did not plan to perform the meta-analysis but instead presented the results using forest plots without pooled estimates (Higgins, 2011). We planned to explore the potential reasons by a subgroup analysis based on the severity of AP and the detailed therapeutic regimen of neostigmine if heterogeneity was found, and there were sufficient relevant data (Higgins, 2011).

### Assessment of publication bias

Funnel plots for measuring the publication bias were performed if there were 10 or more included studies in this meta-analysis, with Egger’s test being used to determine the statistical significance of the publication bias. A *p*-value of less than 0.05 was considered to indicate the statistically significant publication bias (Egger et al., 1997). If fewer than 10 studies were included, the publication bias was assessed based on the characteristics of the included studies instead.

### Data synthesis

Data were analyzed using RevMan (version 5.4.1). A random-effects model was used to combine the eligible trials, in which the DerSimonian and Laird method was used to estimate the between-study variance. The results were presented as forest plots and risk ratios (RRs) with 95% confidence interval (CI) for dichotomous data and mean MD with 95% CI for continuous data. If the data were reported as the median, the minimum and maximum values, and/or the first and third quartiles, we transformed the data to the mean value and standard deviation (SD) to pool the results in a consistent format (Wan et al., 2014; Luo et al., 2018).

## Results

### Search results and study characteristics

**FIGURE 1:**
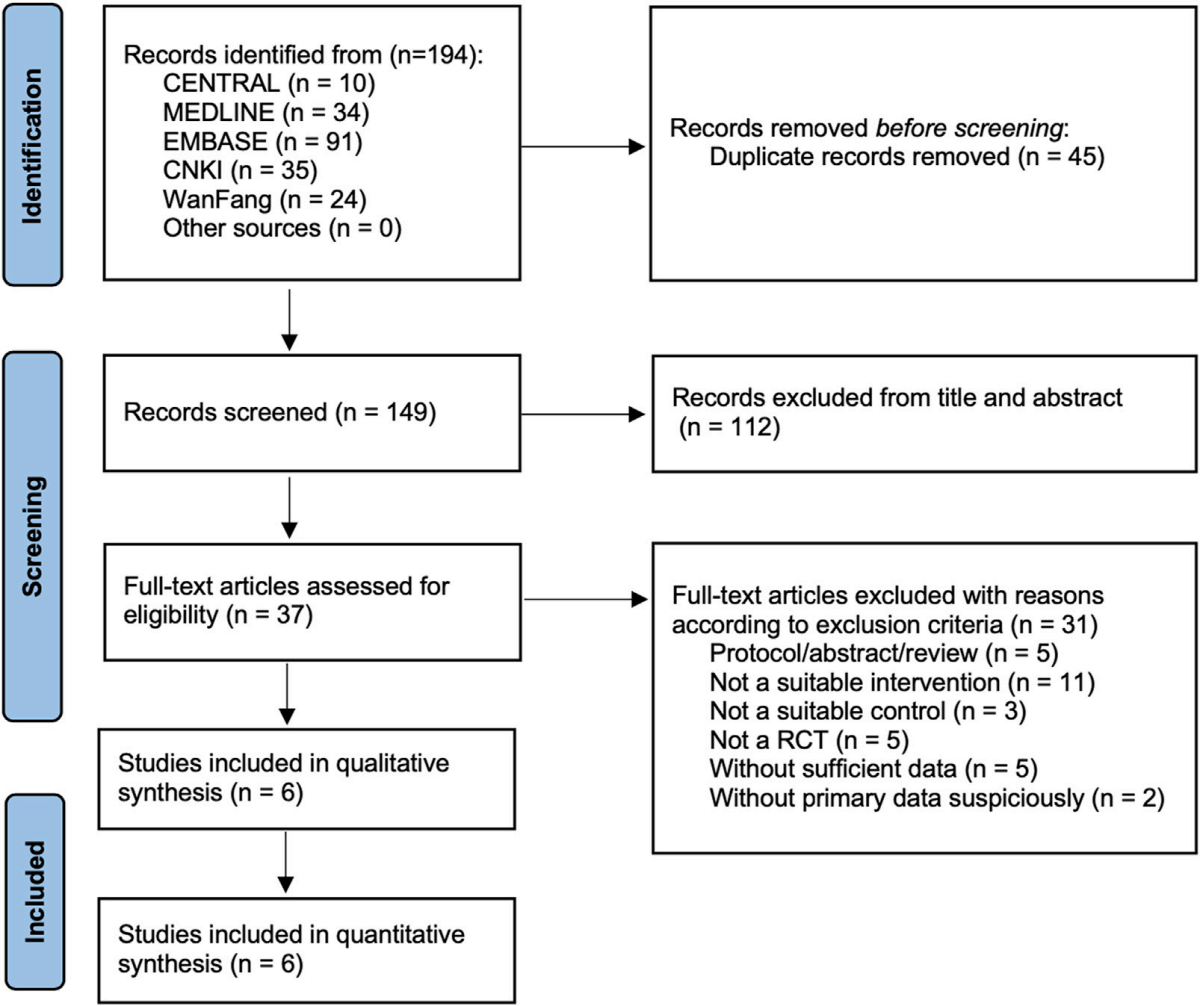
PRISMA flowchart represents the flow of information through the different phases of the systematic review and meta-analysis.

As shown in Figure 1, six RCTs fulfilling all eligible criteria from 194 records were included in this meta-analysis (Feng, 2011; Chen and Zhang, 2013; Wang and Xiao, 2013; Chen, 2016; Wang, 2016; He et al., 2022). Table 1 shows the details of the included studies with a total of 318 participants. A total of 161 participants were in NT, and the remaining 157 were in CT.

**TABLE 1:** Characteristics of the included studies.

First author (publication year)	Patients, n (NT/CT)	Severity of AP	Age, years, and mean ± SD (NT *vs.* CT)	M/F; n (NT *vs.* CT)	Dosage, frequency, and the route of neostigmine	Location of the neostigmine injection	Course of neostigmine treatment (d)	Primary outcomes	Secondary outcomes
Feng (2011)	25/25	SAP	41.0 ± 11.3 *vs.* 39.6 ± 10.6	16/9 *vs.* 14/11	1.0 mg every 12 h; sc	Zusanli	5	①	③⑤⑦
Chen and Zhang (2013)	21/20	SAP	40.1 ± 0.2^*^	26/15^*^	1.0 mg every 6 h; sc	Zusanli	3–5	①②	⑥
Wang and Xiao (2013)	32/28	SAP	45.1 ± 3.0^*^	34/26^*^	1.0 mg every 24 h; sc	Zusanli	7	①②	⑥
Wang (2016)	27/27	SAP	45.2 ± 6.2^*^	30/24^*^	1.0 mg; sc and frequency NM	Zusanli	NM	①②	—
Chen (2016)	16/17	MSAP + SAP	51.4 ± 12.7 *vs.* 54.3 ± 14.8	11/5 *vs.* 13/4	0.5 mg every 12 h; im	—	3–7	—	①②③④⑥⑦
He et al. (2022)	40/40	MSAP + SAP	46.0 ± 13.0 *vs.* 49.0 ± 14.0	27/13 *vs.* 34/6	1.0 mg every 12 h at the beginning and gradually increased to every 6–8 h depending on the response; im	—	7	—	①②③④⑤⑥⑦⑧

Abbreviations: NT, neostigmine treatment group; CT, conventional treatment group; M/F, male/female; *: the clinical data were mentioned in the included study in total rather than in subgroups as NT versus CT and no significant difference between NT and CT; sc, subcutaneously; im, intramuscularly; SD, standard deviation; AP, acute pancreatitis; MSAP, moderately severe AP; SAP, severe AP; ACS, abdominal compartment syndrome; IAP, intra-abdominal pressure; the primary outcomes are as follows: ① time to the first defecation and ② time to the relief of abdominal symptoms. The secondary outcomes are as follows: ① percentage decrease of IAP at 24 h; ② new-onset ACS; ③ in-hospital mortality; ④ multiple organ failure; ⑤ interventional drainage and operation events; ⑥ length of ICU stay; ⑦ length of hospital stay; and ⑧ serious adverse events caused by neostigmine.

### Risk of bias and summary of the main findings

**FIGURE 2:**
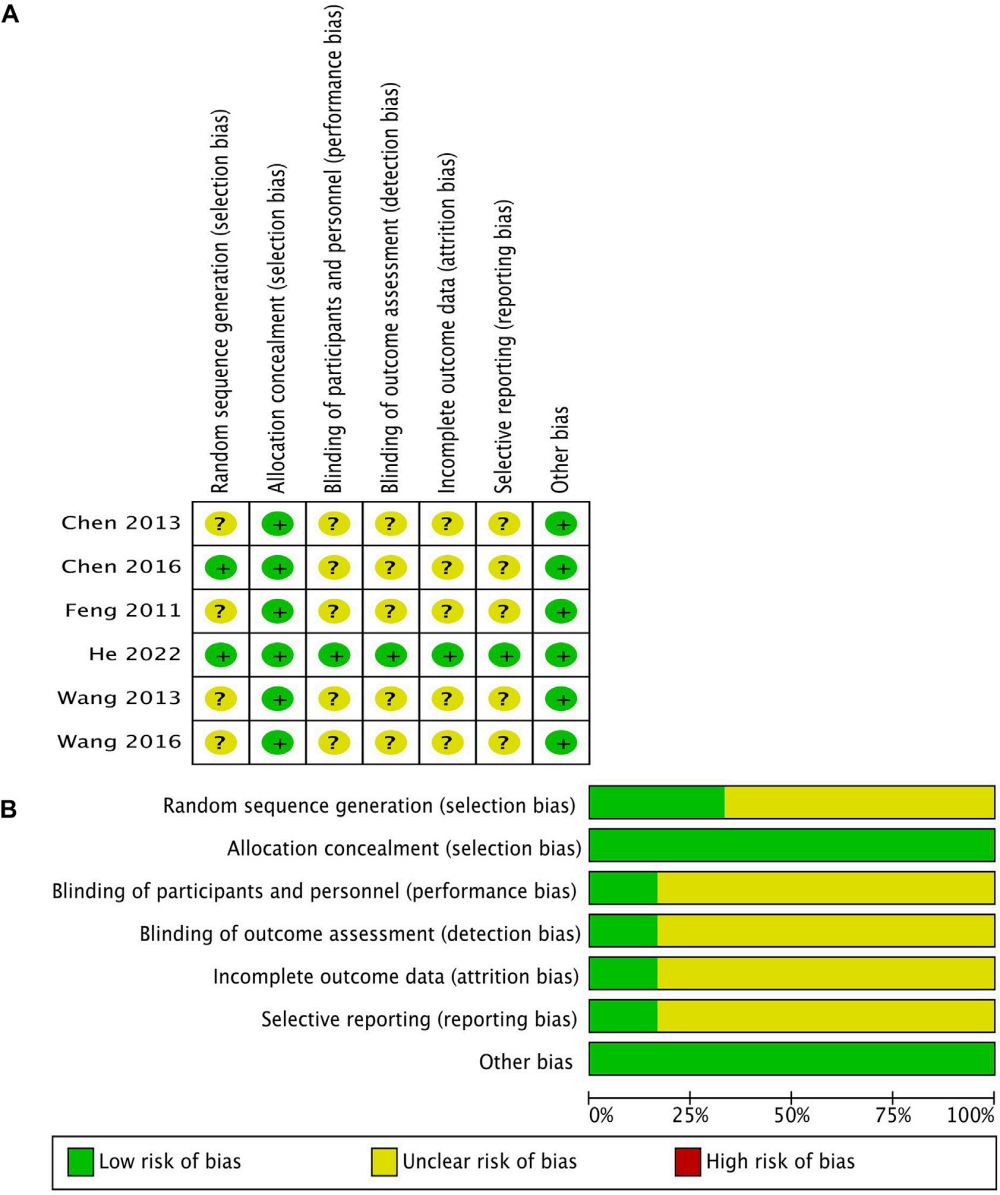
Methodological quality of the included studies according to the Cochrane Collaboration’s tool for assessing the risk of bias. **(A)** Risk of bias summary; **(B)** risk of bias graph.

The risk of bias of eligible studies is shown in Figure 2. All RCTs, except the latest one (He 2022), had some concerns of bias. The quality of evidence for the main outcomes mentioned previously using the GRADE methodology is shown in Table 2.

**TABLE 2:** Summary of the main findings.

Population: Patients with non-mild AP					
Intervention: NT					
Comparison: CT					
Outcome	Anticipated absolute effects^*^ (95% CI)		Relative effect (95% CI)	No. of participants (studies)	Quality of the evidence (GRADE)
	Risk with CT	Risk with NT			
Time to the first defecation	Mean time to the first defecation was 3.42 days	MD −1.74 days (−2.10 to −1.38)	—	205 (four randomized controlled trials)	⊕⊕ ◯ ⃝ Low^ab^
Time to the relief of abdominal symptoms	Mean time to the relief of abdominal symptoms was 3.72 days	MD −1.59 days (−2.07 to −1.11)	—	155 (three randomized controlled trials)	⊕⊕ ◯ ⃝ Low^ab^
Percent decrease of IAP at 24 h	Mean percent decrease of IAP at 24 h was 6.30%	MD −8.95% (−13.95 to −3.95)	—	113 (two randomized controlled trials)	⊕⊕⊕ ◯ Moderate^b^
Length of ICU stay	Mean length of ICU stay was 9.05 days	MD −2.81 days (−3.75 to −1.87)	—	214 (four randomized controlled trials)	⊕⊕⊕ ◯ Moderate^b^

GRADE: working group GRADE of evidence. High quality: further research is very unlikely to change our confidence in the estimate of the effect. Moderate quality: further research is likely to have an important impact on our confidence in the estimate of the effect and may change the estimate. Low quality: further research is very likely to have an important impact on our confidence in the estimate of the effect and is likely to change the estimate. Very low quality: we are very uncertain about the estimate. CI, confidence interval; RR, relative risk. ^*^The basis for the assumed risk is the average control group proportion across all comparisons; AP, acute pancreatitis; NT, neostigmine treatment group; CT, conventional treatment group; MD, mean difference; IAP, intra-abdominal pressure; ^a^downgraded one level for the risk of bias; ^b^downgraded one level for a small sample size.

### Publication bias

We assessed the publication bias on the basis of the characteristics of the included studies rather than the funnel plots because fewer than the 10 included studies were analyzed. We assessed the risk of bias to be unclear because the protocols and registered information on five RCTs in our review were not found despite the fact that we obtained the registered information on the last RCT (He 2022) from the clinical trial registration website (No. NCT02543658).

## Effects of interventions

### Primary outcomes

#### Time to the first defecation

**FIGURE 3:**
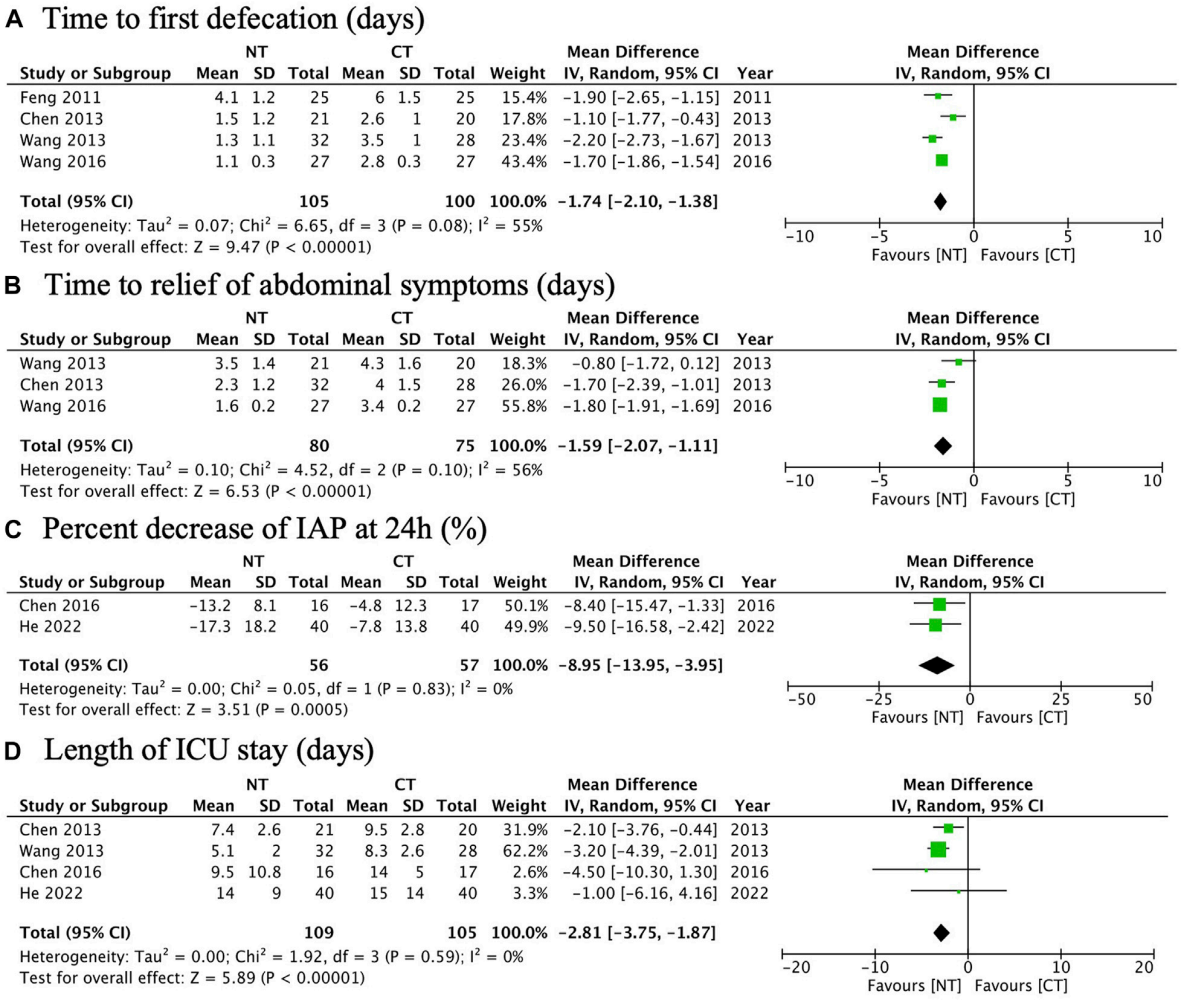
Forest plots illustrating the main outcomes (the primary outcome and some secondary outcomes): **(A)** time to the first defecation; **(B)** time to the relief of abdominal symptoms; **(C)** percentage decrease of IAP at 24 h; **(D)** length of ICU stay.

**FIGURE 4:**
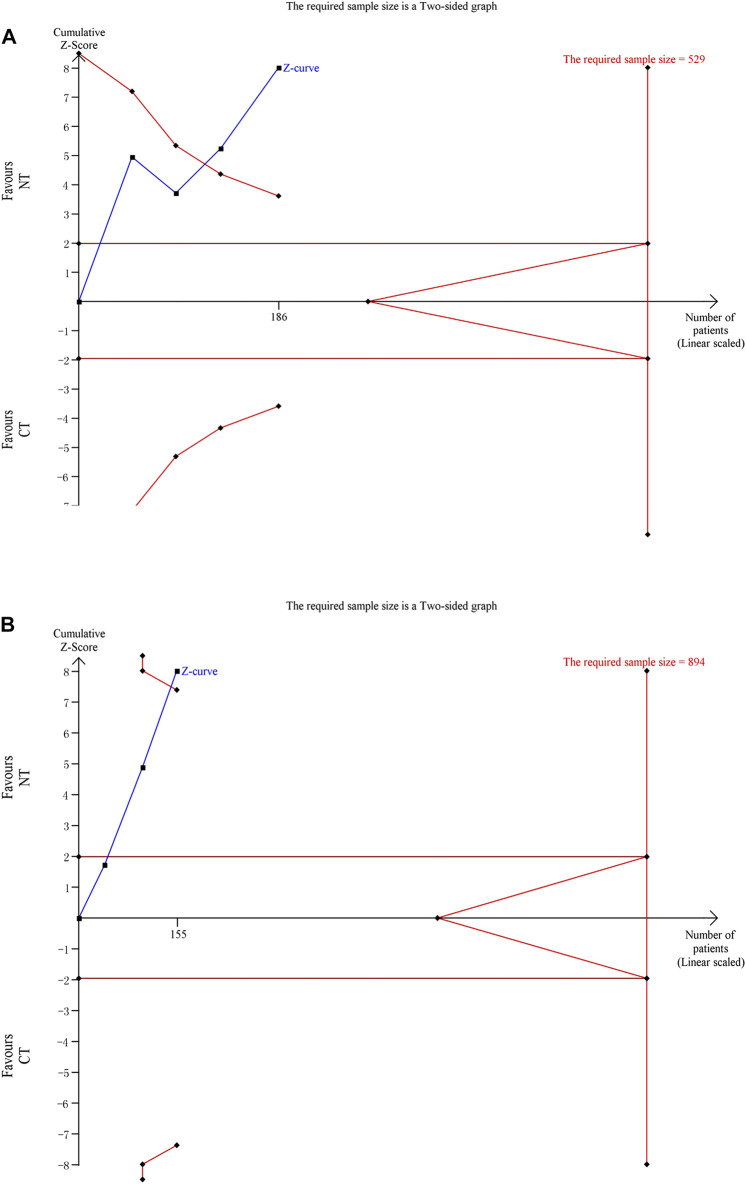
Trial sequential analyses for primary outcomes: **(A)** time to the first defecation and **(B)** time to the relief of abdominal symptoms.

Four RCTs were analyzed for the time to the first defecation. A meta-analysis showed that NT had a significantly shorter time to the first defecation than CT (MD: −1.74; 95% CI: −2.10 to −1.38; *p* < 0.00001; I^2^ 55%; *n* = 205; RCTs = 4; low quality of evidence) (Figure 3A). The TSA result showed the required information size was 529. Although the cumulative Z curve did not reach the required information size, it crossed the conventional boundary and the trial sequential monitoring boundary, suggesting a reliable positive result (Figure 4A).

#### Time to the relief of abdominal symptoms

Three RCTs were analyzed for the time to the relief of abdominal symptoms. A meta-analysis showed that NT had a significantly shorter time to the relief of abdominal symptoms than CT (MD: −1.59; 95% CI: −2.07 to −1.11; *p* < 0.00001; I^2^ 56%; *n* = 155; RCTs = 3; low quality of evidence) (Figure 3B). The TSA result showed the required information size was 894. The cumulative Z curve crossed the conventional boundary and the trial sequential monitoring boundary despite the fact that it did not reach the required information size, suggesting a reliable positive result (Figure 4B).

### Secondary outcomes

#### Percentage decrease of IAP at 24 h

Two RCTs were analyzed for the percentage decrease of IAP at 24 h. A meta-analysis showed that NT had a significantly faster rate of the percentage decrease of IAP at 24 h than CT (MD: −8.95%; 95% CI: −13.95 to −3.95%; *p* = 0.0005; I^2^ 0%; *n* = 113; RCTs = 2; moderate quality of evidence) (Figure 3C).

#### Length of ICU stay

Four RCTs were analyzed for the length of ICU stay. A meta-analysis showed that NT had a significantly shorter length of ICU stay than CT (MD: −2.81; 95% CI: −3.75 to −1.87; *p* < 0.00001; I^2^ 0%; n = 214; RCTs = 4; moderate quality of evidence) (Figure 3D).

#### Serious adverse events caused by neostigmine

There was one RCT that reported the safety outcome of neostigmine as serious adverse events (He 2022). The latest RCT reported six adverse events including circulatory failure, respiratory failure, renal failure, and bradycardia. After careful analyses, the investigators considered neostigmine treatment unlikely to be related to all these adverse events, which were attributed to the progression of AP and the withdrawal of a cardio-selective beta receptor blocker.

#### Other secondary outcomes

**FIGURE 5:**
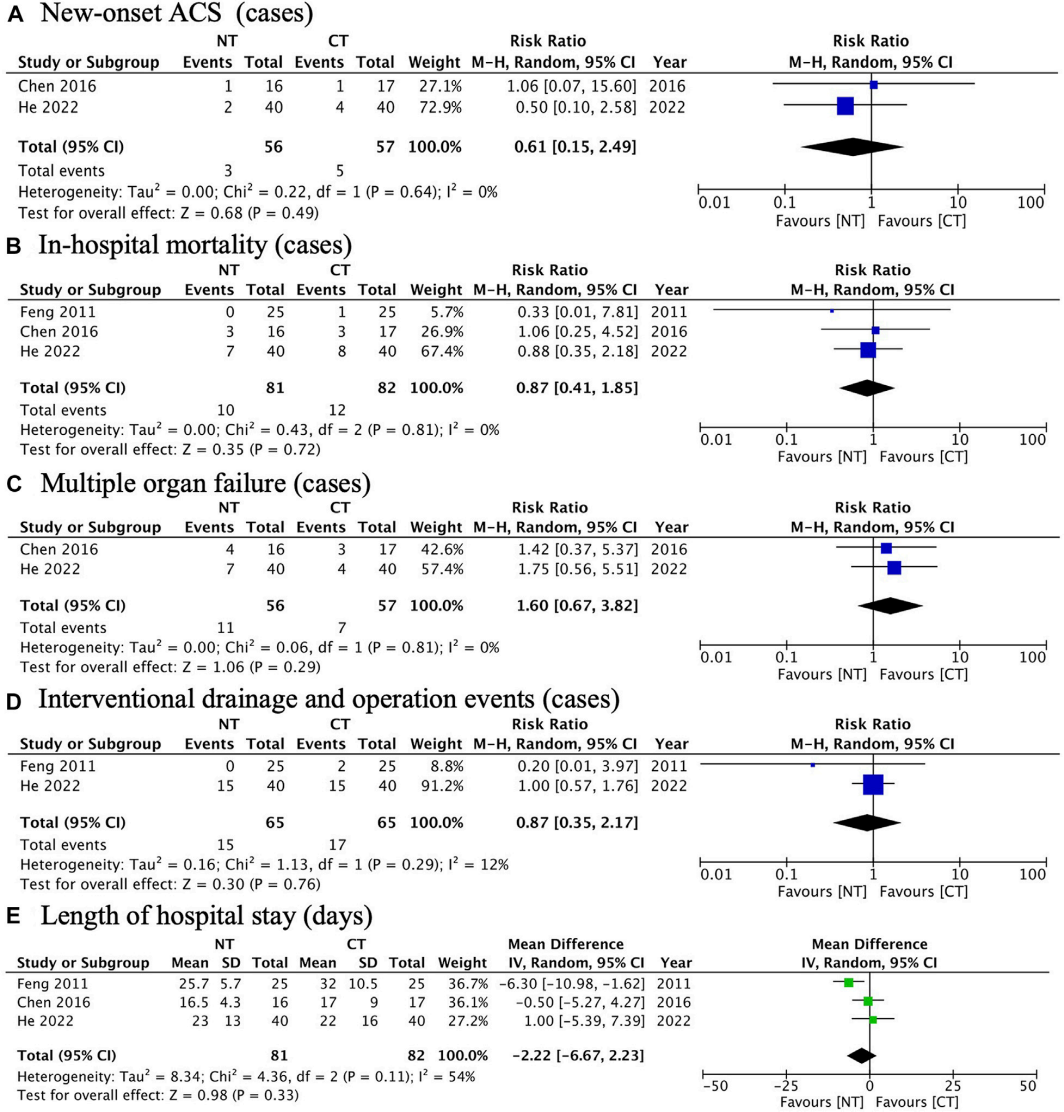
Forest plots illustrating other secondary outcomes: **(A)** new-onset ACS; **(B)** in-hospital mortality; **(C)** multiple organ failure; **(D)** interventional drainage and operation events; **(E)** length of hospital stay.

Meta-analysis showed there was no significant difference between NT and CT in the secondary outcomes, shown as follows:

New-onset ACS (RR: 0.61; 95% CI: 0.15 to 2.49; *p* = 0.49; I^2^ 0%; n = 113; RCTs = 2) (Figure 5A).

In-hospital mortality (RR: 0.87; 95% CI: 0.41 to 1.85; *p* = 0.72; I^2^ 0%; *n* = 163; RCTs = 2) (Figure 5B).

Multiple organ failure (RR: 1.60; 95% CI: 0.67 to 3.82; *p* = 0.29; I^2^ 0%; *n* = 113; RCTs = 2) (Figure 5C).

Interventional drainage and operation events (RR: 1.60; 95% CI: 0.67 to 3.82; *p* = 0.29; I^2^ 0%; n = 130; RCTs = 2) (Figure 5D).

Length of hospital stay (MD: −2.22; 95% CI: −6.67 to 2.23; *p* = 0.33; I^2^ 54%; *n* = 163; RCTs = 3) (Figure 5E).

## Discussion

Our systemic review and meta-analysis showed that compared with the conventional treatment, the application of neostigmine promoted the recovery of bowel movements in patients with non-mild AP and fastened the percentage decrease of IAP at 24 h and shortened the length of ICU stay, suggesting that neostigmine may be beneficial to promote the recovery of the gastrointestinal function and improve clinical prognoses. The TSA showed that the results for primary outcomes were reliable, regardless of the relatively small sample size. The quality of evidence for the main outcomes was low to moderate due to the paucity of the included studies and risk of bias.

Neostigmine is a reversible acetylcholinesterase inhibitor, which increases acetylcholine and stimulates both nicotinic and muscarinic receptors. Its distribution and elimination half-lives are 3.4 and 77 min, respectively. Therefore, neostigmine is usually used to reverse the effects of non-depolarizing muscle relaxants at the end of an operation, showing a dose-dependent effect and ceiling effect (Miller, 1976; Miller and Roderick, 1977). In the field of gastroenterology, it has been proved that neostigmine can induce colonic decompression in pseudo-obstruction and is recommended in the treatment of patients with colonic obstruction and IAH, who show poor response to other measures. It further supports the beneficial effect of neostigmine in non-mild AP on the promotion of gastrointestinal motility and reduction of IAP revealed by our review (Ponec et al., 1999; Kirkpatrick et al., 2013). As for this, there was no significant difference in secondary outcomes as the new-onset ACS, in-hospital mortality, multiple organ failure, interventional drainage, and operation events and the length of hospital stay, we speculated that the reason may be due to the paucity of the included participants, and further large-sample size studies are needed.

However, as for AP, the international clinical guidelines paid less attention to neostigmine despite the fact that neostigmine with or without TCM was widely used in clinical practice in the department of gastroenterology and ICUs in lots of hospitals in China for non-mild AP, especially with enteroparalysis or IAH (Tenner et al., 2013; Greenberg et al., 2016; Crockett et al., 2018a; Crockett et al., 2018b; Leppaniemi et al., 2019). We speculated that the reason was that all previous related studies were published in the Chinese language in the local journals until He et al. published a high-quality RCT in an international journal in 2022 (He et al., 2022; Mancilla Asencio and Berger Fleiszig, 2022). To the best of our knowledge, this systematic review was the first review that included all RCTs with the latest review published in March, 2022, about neostigmine treatment for non-mild AP, providing strong current evidence in this field.

Nevertheless, our study had several limitations. First, the main limitation was the small sample size of the included studies and participants and the risk of bias due to the inappropriate methodology of several included RCTs published in the Chinese language, which limited its clinical application (Wu et al., 2006). However, TSA revealed that the result of primary outcomes seemed to be reliable, regardless of the unsatisfied sample size. Certainly, large-sample, prospective, multi-center RCTs were required to confirm the final results in the future. Second, the lack of sufficient clinical data and the paucity of the included studies made it difficult to perform further statistical analysis, such as the safety of neostigmine treatment, efficacy of neostigmine versus neostigmine and TCM, exploration of the appropriate dosage, frequency, route of administration, location of the injection, and treatment course for neostigmine treatment.

## Conclusion

Our meta-analysis suggested that for patients with non-mild AP, neostigmine promotes the recovery of the gastrointestinal motility and may have positive effects on the clinical prognosis. Future studies such as large-sample prospective multi-center studies are needed for conclusive results and further detailed analyses.

## Data Availability

The original contributions presented in the study are included in the article/Supplementary Material; further inquiries can be directed to the corresponding authors.
