# Supplementary material for: Neostigmine for non-mild acute pancreatitis: A systematic review and meta-analysis of randomized controlled trials
Source: Front Pharmacol. 2023 Feb 28;14:1131974. doi: 10.3389/fphar.2023.1131974 (PMC10011075; doi:10.3389/fphar.2023.1131974)
Supplement: Supplementary file 2 [file Table2.DOCX]

**Search strategies**

**CENTRAL search strategy (via Cochrane Library)**

1. neostigmine or synstigmin or prostigmin or prostigmine or vagostigmin or polstigmine or proserine or prozerin or syntostigmine

2. MeSH descriptor: [Neostigmine] explode all trees

3. 1 or 2

4. MeSH descriptor: [Pancreatitis] explode all trees

5. Pancreatitis

6. 4 or 5

7. 3 and 6

**MEDLINE search strategy (via OvidSP)**

1. exp pancreatitis/

2. pancreatitis.mp.

3. 1 or 2

4. exp neostigmine/

5. (neostigmine or synstigmin or prostigmin or prostigmine or vagostigmin or polstigmine or proserine or prozerin or syntostigmine).ti,ab.

6. 4 or 5

7. 3 and 6

**EMBASE search strategy (via EMBASE)**

1. ‘pancreatitis’:ab,ti

2. ‘pancreatitis’/exp

3. 1 or 2

4. ‘neostigmine’/exp

5. neostigmine:ab,ti OR synstigmin:ab,ti OR prostigmin:ab,ti OR prostigmine:ab,ti OR vagostigmin:ab,ti OR polstigmine:ab,ti OR proserine:ab,ti OR prozerin:ab,ti OR syntostigmine:ab,ti

6. 4 or 5

7. 3 and 6

**CNKI and WanFang database search strategy**

1. 急性胰腺炎

2. 新斯的明或普洛斯的明或普鲁斯的明或普洛色林

3. 1和2
